# Nonthermal ablation of deep brain targets: A simulation study on a large animal model

**DOI:** 10.1118/1.4939809

**Published:** 2016-01-21

**Authors:** Can Barış Top, P. Jason White, Nathan J. McDannold

**Affiliations:** Department of Radiology, Brigham and Women’s Hospital, Harvard Medical School, 221 Longwood Avenue, Boston, Massachusetts 02115

**Keywords:** focused ultrasound, ablation, acoustic cavitation, acoustic simulation, *k*-space, brain

## Abstract

****Purpose**:**

Thermal ablation with transcranial MRI-guided focused ultrasound (FUS) is currently limited to central brain targets because of heating and other beam effects caused by the presence of the skull. Recently, it was shown that it is possible to ablate tissues without depositing thermal energy by driving intravenously administered microbubbles to inertial cavitation using low-duty-cycle burst sonications. A recent study demonstrated that this ablation method could ablate tissue volumes near the skull base in nonhuman primates without thermally damaging the nearby bone. However, blood–brain disruption was observed in the prefocal region, and in some cases, this region contained small areas of tissue damage. The objective of this study was to analyze the experimental model with simulations and to interpret the cause of these effects.

****Methods**:**

The authors simulated prior experiments where nonthermal ablation was performed in the brain in anesthetized rhesus macaques using a 220 kHz clinical prototype transcranial MRI-guided FUS system. Low-duty-cycle sonications were applied at deep brain targets with the ultrasound contrast agent Definity. For simulations, a 3D pseudospectral finite difference time domain tool was used. The effects of shear mode conversion, focal steering, skull aberrations, nonlinear propagation, and the presence of skull base on the pressure field were investigated using acoustic and elastic wave propagation models.

****Results**:**

The simulation results were in agreement with the experimental findings in the prefocal region. In the postfocal region, however, side lobes were predicted by the simulations, but no effects were evident in the experiments. The main beam was not affected by the different simulated scenarios except for a shift of about 1 mm in peak position due to skull aberrations. However, the authors observed differences in the volume, amplitude, and distribution of the side lobes. In the experiments, a single element passive cavitation detector was used to measure the inertial cavitation threshold and to determine the pressure amplitude to use for ablation. Simulations of the detector’s acoustic field suggest that its maximum sensitivity was in the lower part of the main beam, which may have led to excessive exposure levels in the experiments that may have contributed to damage in the prefocal area.

****Conclusions**:**

Overall, these results suggest that case-specific full wave simulations before the procedure can be useful to predict the focal and the prefocal side lobes and the extent of the resulting bioeffects produced by nonthermal ablation. Such simulations can also be used to optimally position passive cavitation detectors. The disagreement between the simulations and the experiments in the postfocal region may have been due to shielding of the ultrasound field due to microbubble activity in the focal region. Future efforts should include the effects of microbubble activity and vascularization on the pressure field.

## 1. INTRODUCTION

Transcranial MRI-guided focused ultrasound (FUS) is an emerging noninvasive alternative to surgery that is being explored for the treatment of brain tumors and other disorders of the central nervous system. Initial studies have focused on using this technique for thermal ablation of tumors^1–3^ and functional neurosurgery.^4–6^ As this method requires large time-averaged acoustic energies to deliver sufficient energy through bone, the use of a low-frequency hemispherical transducer array was proposed to limit local heating of the skull by distributing the power over a large surface and by increasing the gain of the array.^7,8^ Studies have shown that it is possible using these methods to create lesions within a volume of a few cubic centimeters inside the brain. However, skull heating increases as the focus approaches the skull base and peripheral regions, mainly due to shear mode conversions at the skull bone interfaces.^9–12^ This limits the anatomical region in which the method can be applied safely to the central parts of the brain. In addition, reflections within the skull can result in the formation of standing waves, which can deposit acoustic energy at the ultrasound field antinodes.^13^

Tissue ablation can also be achieved mechanically by using microbubble contrast agents.^14–17^ The interaction between microbubbles and an ultrasound field can cause stable or inertial cavitation of microbubbles, depending on the intensity of the applied acoustic pressure. Stable cavitation has been shown to lead to temporary disruption of the blood–brain barrier (BBB),^18–20^ while higher pressure levels that induce inertial cavitation have been shown to cause physical damage to microvessels.^21^ With this approach, microbubbles can be driven using burst sonications at a low-duty-cycle at a level that induces inertial cavitation, which causes vessel damage and ischemic necrosis.^15–17,22^ The ultrasound intensity required for such exposures was found to be at least two orders of magnitude smaller than the required intensity for thermal ablation.^23^ This nonthermal ablation approach is therefore promising, as it can extend the region where FUS therapy can be performed toward the skull base or the periphery while minimizing damage to normal tissues. Since microbubbles are present in the entire brain, the procedure must be carefully controlled by adjusting the ultrasound power level to prevent damage from occurring outside the focal region in side lobes or in the acoustic beam path. To heighten the level of monitoring, passive cavitation detectors (PCDs) can be used for real-time cavitation activity detection and imaging during therapy.^24–29^

The feasibility of nonthermal ablation for deep brain targets was investigated with nonhuman primates (NHP) in a recent study from our group.^23^ Even though the created lesions were in good agreement with the beam characteristics of the therapy device, in some cases, damage was observed outside of the targeted focal region, predominately in the prefocal area, a few millimeters from the edge of the lesion. BBB disruption without other evident damage was also observed in a larger prefocal region with a distinct pattern. We postulated several possible reasons for these prefocal effects: reflections from the skull base; location of targets that lead to beam path effects between the therapy array; excessive exposure levels due to misalignment of the PCDs and the focal region; and aberration due to shear mode conversion. In this study, we used 3D simulations of this experiment to investigate these factors in order to inform further studies leading to the clinical application of this method.

For the simulations, an implementation of a pseudospectral time domain (PSTD) method available as an open source matlab tool (*k*-Wave^30^) was used. PSTD methods can relax the discretization criteria in the conventional finite difference time domain (FDTD) scheme by calculating spatial derivatives in the spectral domain.^31–33^ The pressure field was calculated and compared for both the acoustic^34^ and elastic wave equations.^35^ The simulation results were compared to the contrast-enhanced MRI obtained during the experiments that showed the lesion and BBB disruption in the beam path. The effect of aberration correction on beam patterns was analyzed by using corrected and uncorrected element weightings. Skull base effects were also analyzed by comparing two different simulations, one that included the full skull geometry and one in which the skull base was removed. The PCD sensitivity pattern inside the skull was also simulated. Finally, as the elastic wave solver did not include nonlinear propagation, the effects of nonlinearity were analyzed using the acoustic solver.

## 2. METHODS

### 2.A. Experiments

We simulated experiments described in detail in a previous paper.^23^ Briefly, nonthermal ablation was performed in the brain in anesthetized rhesus macaques using a 220 kHz clinical prototype transcranial MRI-guided FUS system (ExAblate Neuro, InSightec). This system is a 1024-element phased array with a hemisphere geometry (diameter: 30 cm) that is integrated with a 3T clinical MRI (Signa, GE). The phased array was used to electronically steer the focal region away from its geometric focus. It can also be used to correct for aberrations induced by the skull,^2^ but that feature was not used in these experiments.

Experiments were performed in accordance with procedures approved by the Harvard Medical School Institutional Animal Care and Use Committee. The monkey was housed, fed, watered, and provided with environmental enrichment according to U.S. Department of Agriculture (USDA), Office of Laboratory Animal Welfare (OLAW), and Association for Assessment and Accreditation of Laboratory Care (AAALAC) regulations.

**FIG. 1.**
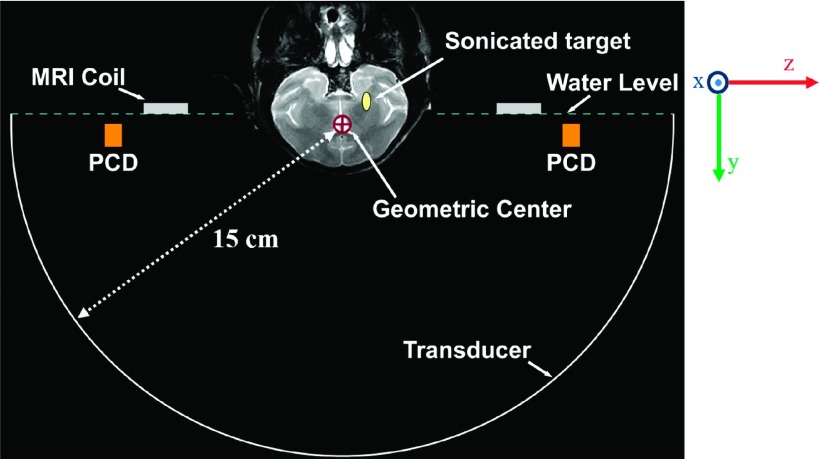
Coronal T2-weighted MR image of a macaque superimposed on a diagram of the experimental setup drawn approximately to scale. The locations of the MRI coil and PCD’s are indicated. The phased array transducer was used to electronically steer the focal point from the geometric center of the FUS array to a target near the skull base.

The experimental results from animal number 2 and 3 in our previous study^23^ were simulated here. After obtaining planning MRI, low-duty-cycle sonications (10 ms bursts, 1% duty cycle, 300 s duration) were targeted at locations near the skull base next to the optic tract. Each sonication was preceded by an intravenous injection of the ultrasound contrast agent Definity (Lantheus) administered at a dose of 20 μl/kg. A diagram of the experimental apparatus is shown in Fig. 1.

The acoustic pressure amplitude used for ablation was selected based on recordings obtained with two PCD’s mounted inside the hemisphere transducer on both sides of the head at a distance of ±10 cm from the geometric focal point. These PCD’s each consisted of a 40 × 7 mm air-backed rectangular PZT element that was weakly focused (radius of curvature 15 cm) and that had a resonant frequency of 610 ± 20 kHz. Activity detected at the resonant frequency of the PCD’s was assumed to be broadband emission, which is created during inertial cavitation.^36–38^ At each target, sonications at increasing pressure amplitudes were made until such activity was observed. The ablation was then performed at exposure levels that were above this threshold. We also quantified harmonic, subharmonic, and ultraharmonic emissions.

After sonication, MRI was obtained to visualize the effects of the sonications. The hemorrhagic lesion produced in the focal region was visualized using T2*-weighted imaging. Contrast-enhanced T1-weighted imaging was used to visualize BBB disruption. This disruption was observed along the ultrasound beam path in the prefocal area. Histological examination found some tissue damage within the area where BBB disruption was observed, predominately in the near-field just below the focal plane. Damage was also observed in the focal plane 1–2 mm from the edge of the lesion. A CT scan (Ceretom) of the animal was also obtained and reconstructed using a bone kernel (voxel size: 0.25 × 0.25 × 0.4 mm).

### 2.B. Simulations

Simulations were performed using a 3D pseudospectral finite difference time domain method (*k*-Wave, matlab toolbox^30^) that solve the discretized wave equations for acoustic or elastic wave propagation on a finite difference grid. At each time step, the fields are transformed to the spectral domain; derivatives are calculated and inverse transformed to the spatial domain. This method is global in the sense that the entire simulation domain is used in approximating the derivative of a single point. The accuracy of the method is, therefore, higher than the conventional high-order finite difference time domain methods, making it possible to discretize the domain in the Nyquist limit (*λ*/2) theoretically for a lossless and homogeneous medium.

The acoustic wave equations used in the acoustic solver were

$$\frac{\partial u}{\partial t}=-\frac{1}{\rho_{0}}\nabla p+S_{F},$$ (1)

$$\frac{\partial \rho }{\partial t}=-\left(2\rho +\rho_{0}\right)\nabla \cdot u-u\cdot \nabla \rho +S_{M},$$ (2)

$$p=c_{0}{}^{2}\left(\rho +d\cdot \nabla \rho_{0}+\frac{B}{2A}\frac{\rho^{2}}{\rho_{0}}+L\rho \right),$$ (3)

where *u*, *d*, *p*, *ρ*_0_, and *ρ* are the particle velocity, particle displacement, pressure, ambient density, and acoustic density perturbation, respectively; *c*_0_ is the speed of sound, *B*/*A* is the nonlinearity parameter, and *L* is an acoustic absorption and dispersion operator.^34^ *S_F_* and *S_M_* are the body force per unit mass and the time rate of input mass per unit volume, respectively.

The visco-elastic wave equations used in the elastic solver were

$$\frac{\partial \sigma_{ij}}{\partial t}=\lambda \delta_{ij}\frac{\partial v_{k}}{\partial x_{k}}+\mu \left(\frac{\partial v_{i}}{\partial x_{j}}+\frac{\partial v_{j}}{\partial x_{i}}\right)+\chi \delta_{ij}\frac{\partial^{2}v_{k}}{\partial x_{k}\partial t}+\eta \left(\frac{\partial^{2}v_{i}}{\partial x_{j}\partial t}+\frac{\partial^{2}v_{j}}{\partial x_{i}\partial t}\right),$$ (4)

$$\frac{\partial v_{i}}{\partial t}=\frac{1}{\rho_{0}}\frac{\partial \sigma_{ij}}{\partial x_{j}}+f_{i},$$ (5)

where *σ_ij_* and *v*_*i*,*j*,*k*_ are the stress and velocity terms, respectively; *λ* and *μ* are the first and second Lamé constants; *χ* and *η* are the compressional and shear viscosity coefficients, respectively. The discretization of acoustic and elastic wave equations and the details of the method can be found elsewhere.^34,35^ The viscoelastic parameters (*λ*, *μ*; *χ*, *η*) were calculated from the compressional and shear sound speed and absorption, and density, derived from the CT images as explained below, using the following relations:^39^

$$\mu =c_{s}{}^{2}\rho ,\;\;\;\;\lambda +2\mu =c_{p}{}^{2}\rho ,$$ (6)

$$\eta =2\rho_{0}c_{s}{}^{3}\alpha_{s},\;\;\;\chi +2\eta =2\rho_{0}c_{c}{}^{3}\alpha_{p},$$ (7)

where *c_s_*, *c_p_*, *α_s_*, and *α_p_* are the shear sound speed, compressional sound speed, shear absorption, and compressional absorption, respectively.

The elastic wave model includes the effect of shear mode conversion and absorption, which is not the case in the acoustic model. Shear modes may be generated inside the skull if the incident wave angle is higher than a critical angle.^12^ While this phenomenon can be better modeled using elastic wave simulations, the computational burden is higher than the acoustic wave model, since the number of field quantities (stress tensor compared to scalar pressure field) and intrinsic parameters (shear mode parameters) are increased. We used both simulation methods to compare the effect of shear mode conversion and absorption on the simulated targets.

The simulation model was formed using MR and CT images of the sonicated animal and the water-filled FUS array. CT images were registered to the MR images of the NHP head and the FUS array using 3D slicer ver.4.4.0.^40^ Registration was done in two steps. In the first step, a coarse linear manual transformation was performed. In the second step, a rigid registration was done using the expert automated registration module, which is based on intensity similarities in a selected region of interest (ROI). The ROI was selected to include fine details of MRI and CT images, which were verified by visual inspection after the registration. After registration, the MRI and CT data of the NHP head were resampled at 1-mm resolution on the same volume with the FUS array MR image. Then, all images were exported as new DICOM image series and imported into matlab for modeling the simulation geometry.

The material parameters used in the simulations were extracted from the CT images. The brain tissue was assumed to be homogeneous, since the ultrasound reflections in soft tissues are small (<1%). The skull was extracted manually using a threshold for the intensity value. The image data were converted to Hounsfield units to determine the porosity of the skull.^41^ Then, the acoustical parameters were calculated from this data assuming a linear relationship between skull porosity and the acoustic parameters.^41,42^ Since a proper data for shear wave parameters were not found as a function of porosity or density, they were set proportional to the compressional parameters.^11,13^ This assumption may affect the elastic wave simulation results, especially in the sidelobe regions where the field strength is relatively low compared to main beam.

The modeling method used here may introduce uncertainties in the simulation results. Nevertheless, this approach has been successfully used for both forward problem and aberration correction simulations. In addition, the skull thickness was relatively small compared to the acoustic wavelength in this study. Although we do not expect a modeling error in the main beam region, the pressure field in the sidelobe regions is more likely to be affected. Nevertheless, the comparison between simulations and experiments show that the uncertainties in our model are not so large as to affect the results and conclusions of this study.

The porosity of the skull was calculated using CT Hounsfield units (*H*) using^41^

$$\psi =1-\frac{H}{1000}.$$ (8)

The speed of sound, absorption, and density of the skull were calculated using^41,42^

$$c_{\mathrm{skull},c}=c_{\mathrm{water}}\;\psi +c_{\mathrm{bone},c}\left(1-\psi \right),$$ (9)

$$\alpha_{\mathrm{skull},c}=\alpha \;\min_{\mathrm{skull},c}+\left(\alpha \;\max_{\mathrm{skull},c}-\alpha \;\min_{\mathrm{skull},c}\right)\psi^{0.5},$$ (10)

$$\rho_{\mathrm{skull}}=\rho_{\mathrm{water}}\;\psi +\rho_{\mathrm{bone}}\left(1-\psi \right),$$ (11)

where *c*_skull,*c*_ and *α*_skull,*c*_ are the compressional sound speed and absorption. The shear wave attenuation $\left(\alpha_{\mathrm{skull},s}\right)$ was set as (90/85) *α*_skull,*c*_, and the shear wave speed *c*_skull,*s*_ was set as (4/7) *c*_skull,*c*_.^11,13^ *ρ*_skull_ is the density of the skull.

The parameters used in Eqs. (9)–(11) and the brain tissue^42–46^ are given in Table I.

Because of the small size of the NHP skull (∼7 cm diameter) compared to the FUS array diameter (30 cm), there was a large distance between the array elements and the skull. To decrease the simulation domain dimensions, a fictitious half sphere (11-cm diameter) surface source was used according to Huygens’s principle.^47^ The surface was placed as close as possible to the skull. The array elements were modeled as circular pistons radiating onto this surface using the Rayleigh integral.^48^ The total pressure amplitude and phase on the surface was obtained by superposing the complex pressure fields of all the elements. A geometric optics approach was used for determining the individual transmission cross sections at the half sphere for each element in the array.

The accuracy of the *k*-space method decreases in inhomogeneous medium.^34^ In order to decide on the required discretization, a simulation study was conducted for which the error in transmission coefficient of a plane wave propagating from water to bone was analyzed. For the bone parameters, maximum possible speed of sound (3100 m/s) and density (2200 kg/m^3^) was used in order to obtain the result for the worst case scenario in terms of impedance mismatch between two media. Consequently, the discretization in the simulations was chosen to be 1 mm (*λ*_water_/6.8) as a compromise between computational burden and accuracy, which resulted in an error below 1.6%. The simulation domain was meshed with 120 × 130 × 120 (*N_x_* × *N_y_* × *N_z_*) cubic cells. A 220-kHz sinusoidal waveform was applied to the array elements for a total simulation duration of 120 μs, which correspond to 18 cm propagation distance (about 3 times the skull length). Element phases were adjusted to steer the focus toward the sonication point without aberration correction in all but one case in which the effect of aberration correction was investigated. The maximum value of the pressure field was recorded for each point in the simulation domain. The total simulation time was 15 min for the acoustic wave model and 35 min for the elastic wave model on a 64-bit computer with a 2.5-GHz dual core CPU and 16-GB RAM.

Before using the reduced model with the fictitious hemispherical source, the reduction method was verified by comparing results with those of the original simulation model. The original model included the entire FUS array and consisted of 324 × 230 × 324 (*N_x_* × *N_y_* × *N_z_*) cubic cells.

We also simulated the field produced by the PCD at its resonant frequency (610 kHz) and at the subharmonic of the FUS system (110 kHz); the sensitivity pattern is proportional to the transmitted field. A significant increase in subharmonic emission occurs at a pressure slightly below the inertial cavitation threshold,^49^ and due to its lower frequency and reduced absorption from the skull, it may be a more sensitive signature to guide nonthermal ablation. In the simulation model, a 40 × 7 mm PCD was placed 10 cm away from the geometric focus of the FUS array as in the case in experiments. For the 610 kHz simulation, the mesh size was 0.35 mm (∼*λ*/7), whereas it was 1 mm (∼*λ*/14) for the 110 kHz simulation. The simulation time increased to 150 μs (27 cm propagation distance) to compensate for the PCD distance. To understand what part of the field of FUS array was likely to dominate the signal received by the PCD, we multiplied their respective normalized pressure fields. We made the assumption that the radiated pressure from the bubbles was linearly correlated with the applied acoustic pressure, a condition that does not apply to nonlinear activity of cavitating bubbles. Notwithstanding this assumption, the combined PCD+FUS intensity pattern should give a first-order indication of the sensitivity map.

The left hemisphere sonication target for animal number 3 was simulated throughout the study in order to compare the simulation results for different scenarios. The volumes of the regions where the pressure amplitude was greater than −3.5, −7, −12, and −14 dB relative to the beam peak were calculated; the former two levels were used as a measure of the main beam volume, whereas latter two were used as a measure of the extent of the side lobe region. The −3.5 and −14 dB levels were chosen as they were found to approximate the boundaries of the created lesion and BBB disruption in the experimental study for the specified target, respectively.

Elastic wave simulations were conducted to compare the MRI findings with the pressure field distribution obtained using simulations for the Animals 2 and 3 (two targets for each animal). An approximate pressure threshold for BBB disruption and necrosis were calculated using these results. CT scans were not available for Animal 1. MR images in Animal 4 were severely distorted due to the presence of a metallic pellet in the brain. Sonications in those animals were thus not simulated.

## 3. RESULTS

### 3.A. Acoustic/elastic model

**FIG. 2.**
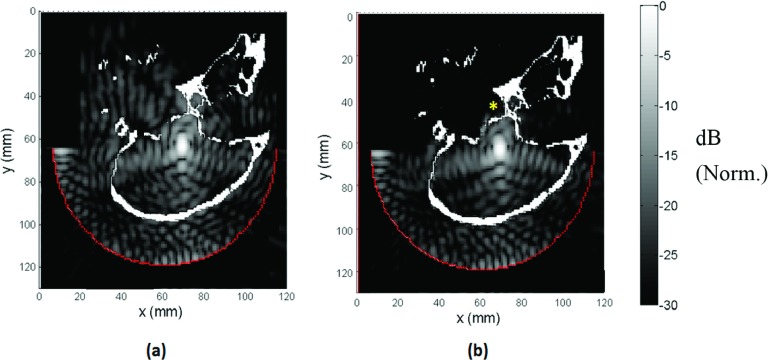
Pressure fields obtained using (a) acoustic and (b) elastic wave simulations (sagittal view). To decrease the simulation domain dimensions, a fictitious 11 cm diameter hemispherical surface source closer to the skull was used in which the simulated array elements radiated using the Rayleigh integral. Pressure field was lower near skull base in the elastic simulation compared to the acoustic simulation case (*). Norm.: Normalized.

**FIG. 3.**
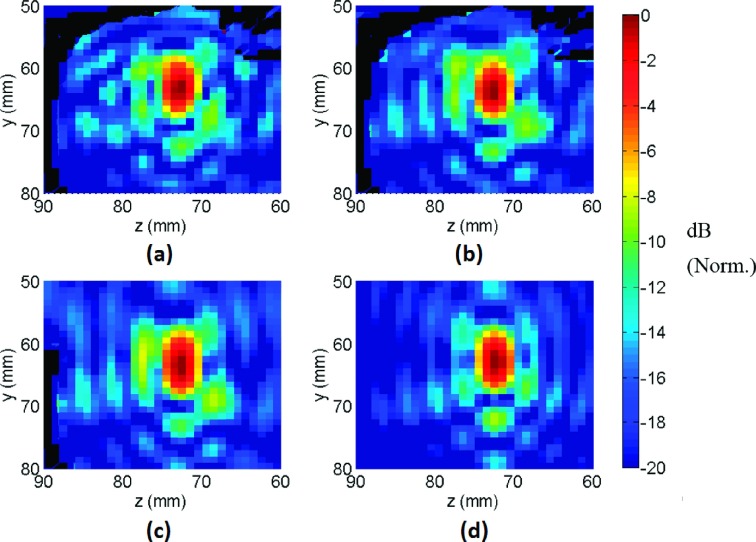
Coronal view showing simulated pressure fields for (a) acoustic wave simulation with the full NHP model, (b) elastic wave simulation with the full NHP model, (c) elastic wave simulation with the skull base removed from the NHP model, and (d) elastic wave simulation without the NHP model. Norm.: Normalized.

The results of the acoustic and elastic simulation models are shown in Fig. 2. The added absorption with shear mode conversion was reflected in the field maps as generalized and local areas of decreased pressure, especially near the skull base (* in Fig. 2), when the elastic model was used. The beam shape was similar for both models, except for some minor differences in the side lobe region (Fig. 3). The pressure around the focal point (±15 mm) was interpolated to obtain 0.1 mm resolution in all dimensions and the normalized peak pressure and the distortion of the field around the focal point were calculated using the interpolated pressure field (Table II).

**FIG. 4.**
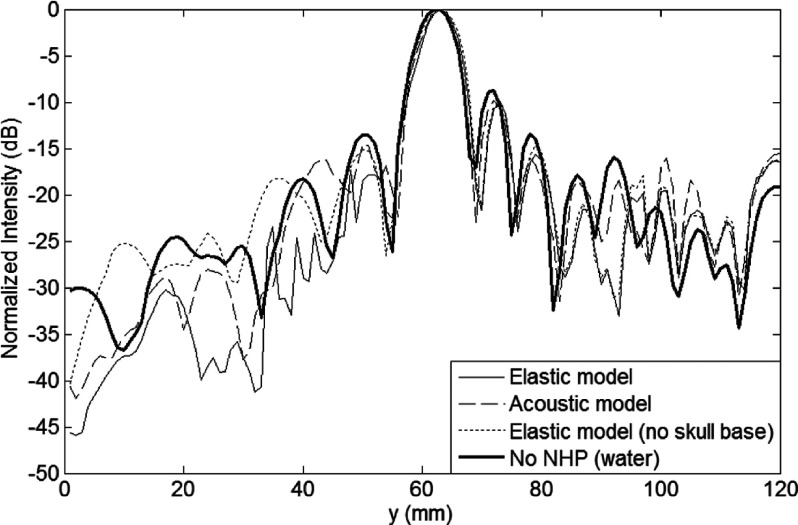
Axial beam plots obtained using the acoustic and elastic wave simulations with full NHP model, elastic wave simulation with the skull base removed from the NHP model, and elastic wave simulation without the NHP model.

The presence of the skull reduced the peak pressure amplitude by about 32% for the acoustic model and 40% for the elastic model. The peak pressure in the elastic simulation was lower by 11% compared to the acoustic model, which may be due to the differences caused by the presence or absence of shear mode propagation. When compared to simulations of the field without the skull, the main beam was distorted with a 1-mm shift of the pressure peak in the prefocal direction along the transducer axis (Fig. 4) for the elastic simulation model. The shift was slightly smaller in the acoustic model. The main beam region volume (i.e., volumes contained by −3.5 and −7 dB iso-pressure contours) was not affected by the simulation type. The side lobe region above −12 dB was slightly larger in the elastic simulation compared to the acoustic simulation, whereas region above −14 dB was larger in the acoustic simulation. In the presence of NHP, the volumes above the −12 and −14 dB thresholds increased by ∼30%–40%.

### 3.B. Comparison of simulations and experiments

**FIG. 5.**
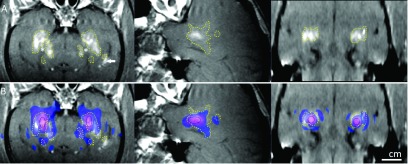
Simulation results (elastic wave model) superimposed on contrast-enhanced T1-weighted contrast-enhanced images acquired shortly after sonication (left: coronal view; middle: sagittal view for the left hemisphere target; right: axial view in the focal plane) for Monkey 3. (A) Hyperintense regions show the disruption of the BBB. The extent of the disruption was manually segmented (dotted lines). BBB disruption was not observed in white matter (*). Greater signal enhancement was observed in a ventricle that was in the beam path (arrow). (B) The simulated pressure field was thresholded at −14 dB for the left hemisphere target, −15 dB for the right hemisphere target, and superimposed on the MRI as a colored region. The extent of the BBB disruption was consistent with the simulated side lobes in the prefocal region, but not in the postfocal region. The black circle indicates the −3.5 dB contour of the simulation, which matched the size of the lesion seen in T2*-weighted MRI.

The simulation results of the elastic model were registered to the contrast-enhanced T1-weighted images obtained in the experiments. These images show the BBB disruption produced in the prefocal region as hyperintense regions. For animal number 3, the simulated beam pattern, when thresholded to approximately match the region with BBB disruption, was cut at −14 and −15 dB with respect to its peak, for the right and left sonication targets, respectively (Fig. 5). These thresholds suggest that BBB disruption occurred at a lower pressure limit of 83 kPa (right target) and 74 kPa (left target), as the peak pressure was 415 kPa in the experiments.^23^ In general, the beam pattern fit well to the hyperintense regions. Side lobe peaks evident in the prefocal region of the simulation were consistent with the regions where BBB disruption was observed in MRI. However, BBB disruption was not observed at the side lobes in the postfocal region. The simulation results were also compared with the lesion size in the T2*-weighted images. It was found that the lesion size coincided best with a −3.5 dB contour of the normalized pressure (data not shown), which corresponded to a pressure of 277 kPa.

**FIG. 6.**
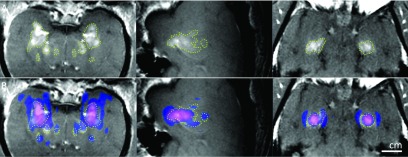
Simulation results (elastic wave model) superimposed on contrast-enhanced T1-weighted contrast-enhanced images acquired shortly after sonication (left: coronal view; middle: sagittal view for the left hemisphere target; right: axial view in the focal plane) for Monkey 2. (A) Hyperintense regions show the disruption of the BBB. The extent of the disruption was manually segmented (dotted lines). BBB disruption was not observed in white matter (*). (B) The simulated pressure field was thresholded at −14 dB for the left hemisphere target, −14.6 dB for the right hemisphere target, and superimposed on the MRI as a colored region. The extent of the BBB disruption was consistent with the simulated side lobes in the prefocal region, but not in the postfocal region.

Similarly, for the animal number 2, the simulated beam pattern was cut at −14 and −14.6 dB levels with respect to its peak, for the right and left sonication targets, respectively (Fig. 6). These thresholds suggest that BBB disruption occurred at a lower pressure limit of 99 kPa (right target) and 92.3 kPa (left target), as the peak pressure was 496 kPa in the experiments.^23^ As in the third animal, the BBB disruption was not observed in the postfocal region. The beam pattern matches well with the hyperintense regions.

### 3.C. Effect of aberration correction

**FIG. 7.**
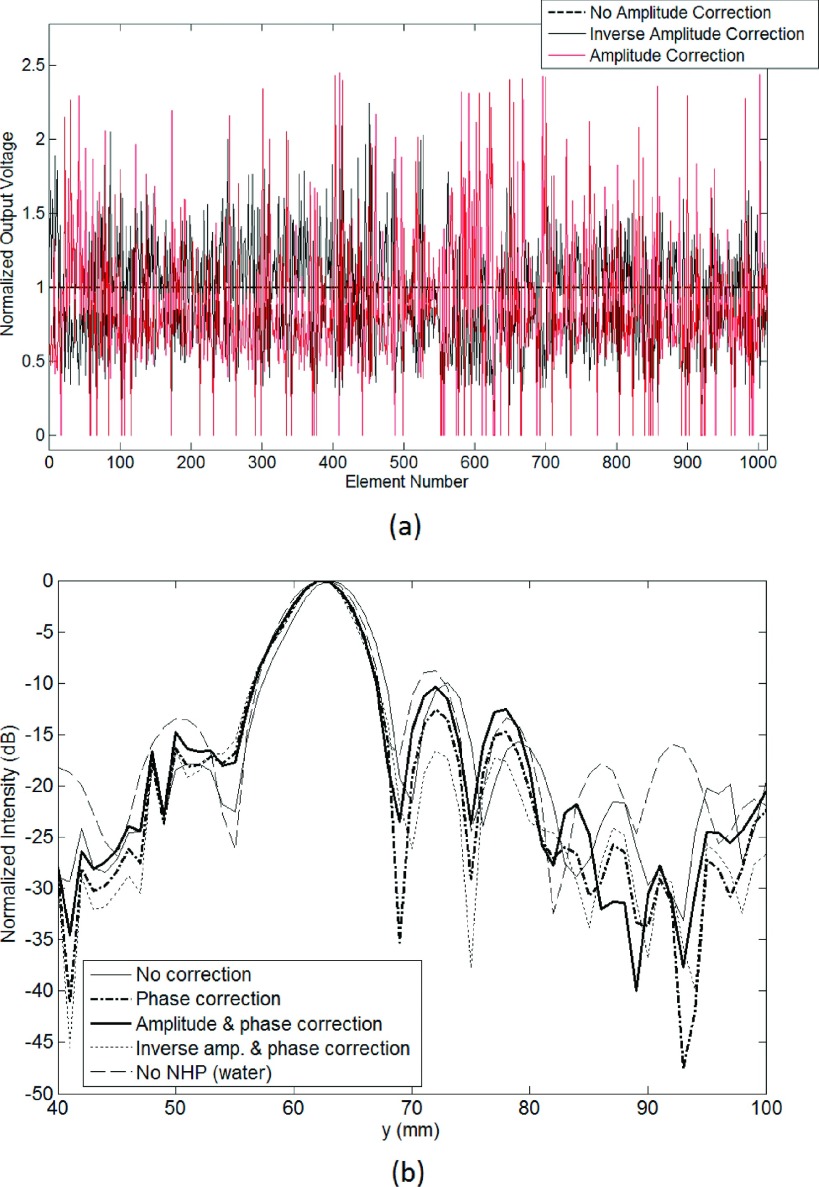
(a) Individual element voltages for amplitude correction and inverse amplitude correction. (b) Axial beam patterns for different aberration correction schemes.

The effect of phase and amplitude aberration corrections induced by the NHP skull was investigated with simulations. The corrections were found by simulating a point source placed at the focal point and finding the phase and amplitude of the received signal at the center of each element. Three different aberration correction methods were used: phase-only, phase and amplitude, phase and inverse amplitude.^50^ The element amplitudes were normalized so that the total output power was constant in each case. With phase and amplitude correction, the amplitudes of the elements were adjusted so that the radiated pressure amplitudes from each element were equal at the focal point. In this case, some of the transducers required a very high excitation coefficient (approaching up to 17 times higher than the mean excitation coefficient). In order to distribute the total power more evenly to all of the transducers, elements that required excitation coefficient higher than 2 times the mean excitation coefficient (*n* = 67) were excluded. In the phase and inverse amplitude correction case, the radiated pressure amplitudes from each element were adjusted to proportionally increase the output of elements that transmitted through skull areas of lower attenuation. The normalized element voltages are plotted in Fig. 7(a) for the cases with amplitude correction. Figure 7(b) shows the axial beam plots for no correction, phase correction, phase and amplitude correction, and phase and inverse amplitude correction simulations. The peak pressures for the different type of aberration correction methods are listed in Table III, along with the volumes above −3.5 and −14 dB relative to the beam peak.

The results showed that the main beam was largely unaffected by the propagation through the NHP skull, except for a 1-mm shift in the beam peak position that could be corrected by adjusting the phases of the elements. Compared to having no correction, phase-only and phase and inverse amplitude correction increased the peak pressure value by 5% and 10%, respectively. It decreased by 15% for the phase and amplitude corrected case.

**FIG. 8.**
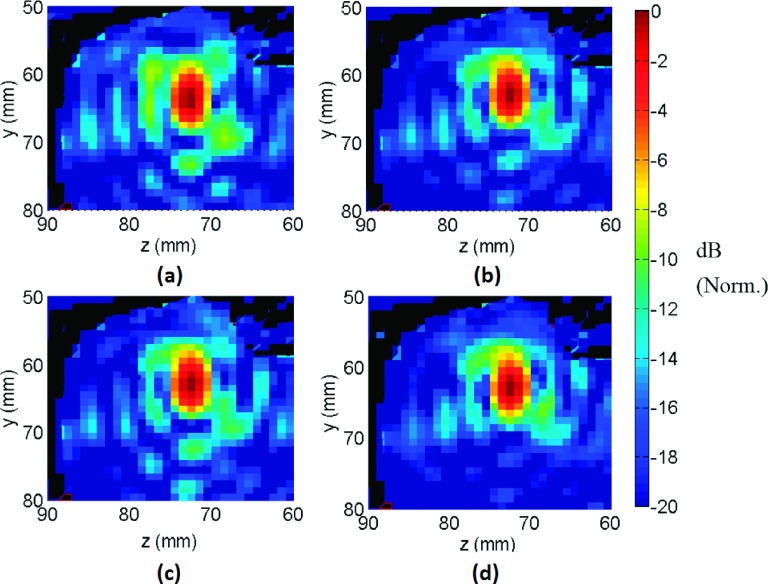
Coronal view showing pressure field obtained using simulations (a) without correction, (b) phase-only correction, (c) amplitude and phase correction, and (d) inverse amplitude and phase correction. Norm.: Normalized.

Examination of the beam plots in the coronal plane revealed differences in the side lobe levels for three types of aberration correction (Fig. 8). The phase and amplitude correction scheme did not introduce any additional advantages (e.g., lowered side lobes) over phase-only correction in terms of beam pattern. However, phase and inverse amplitude correction resulted in a higher peak pressure amplitude and a decreased side lobe volume.

### 3.D. The effect of the skull base

The effect of the skull base on the ultrasound field was investigated by comparing the field with and without its inclusion in the simulation. The peak pressure level was similar for both cases, but the main beam volume (volume above −3.5 dB) increased by ∼20% when the skull base was not present (Table II). The resulting pressure field exhibited only minor differences in the first side lobe region between the focal point and the skull base (compare Fig. 3 top-right and bottom-left), suggesting that skull base reflections were not significant.

### 3.E. Nonlinear effects

To investigate nonlinear effects during these experiments, a simulation with a nonlinear acoustic model was performed, and the results were compared with the linear acoustic model results. The *B*/*A* parameter was set to 7.1 (Ref. 51) for the tissue and skull in the nonlinear model, and 5.2 for water. The maximum pressure at the focus was 561 kPa in the simulations, which was the maximum pressure used in the experimental study.^23^ In order to decrease the bandwidth of the fundamental component for resolving harmonic components, the simulation duration was increased to 360 μs in this analysis. There was no evident difference in the pressure field maps generated by the linear and the nonlinear models. The time domain waveform at the focal point was transformed to the frequency domain to analyze the amplitude of the harmonic components in the nonlinear model. The second harmonic was found to be 48 dB below the fundamental component. The finite duration of the simulation excluded the third harmonic from being resolved in the frequency plot.

### 3.F. The effect of focal steering

**FIG. 9.**
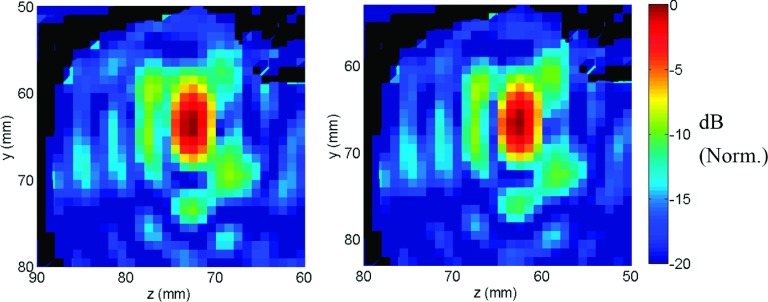
Coronal view showing pressure field obtained using simulations in its original steered position (left) and for the case in which the target was moved to the geometrical center (right) of the hemispherical array. Norm.: Normalized.

In the experiment, the focus was steered 16 mm in the lateral direction and 3 mm in the axial direction. In order to examine the effects of beam steering, a simulation was conducted with the skull artificially relocated so that the target was at the center of the array (i.e., no applied steering). The maximum pressure level at the focus was increased by 13% in this case. The side lobe pattern (Fig. 9) was similar to the steered case with a 12% decrease in the volume with intensity level greater than −14 dB relative to beam peak (Table III).

### 3.G. PCD sensitivity

**FIG. 10.**
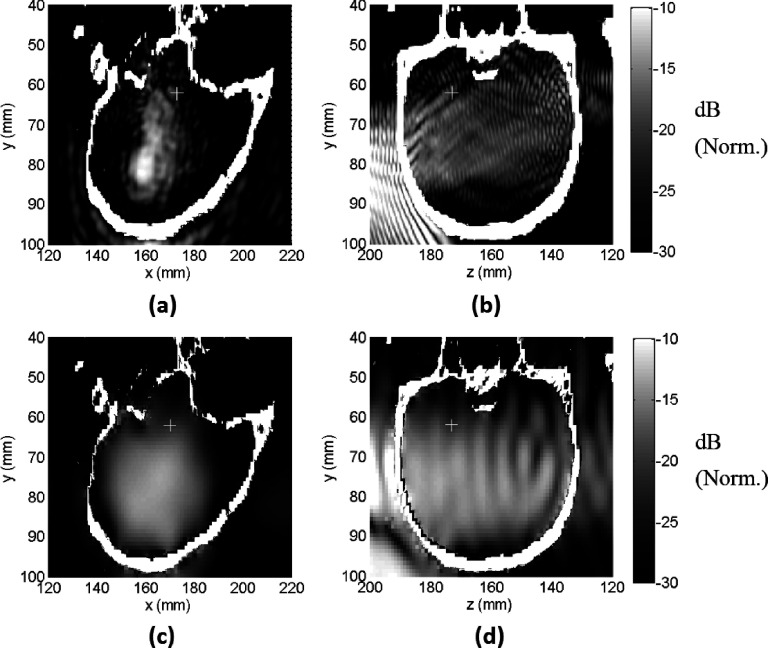
Pressure field radiated by one of the PCD’s used in the experiments. The receive field is proportional to the transmit field. [(a) and (b)] Simulated field at 610 kHz; [(c) and (d)] simulated field at 110 kHz (half the frequency of the FUS device). The focal point is shown in the plots (+). [(a) and (c): sagittal view; (b) and (d): coronal view.] Norm.: Normalized.

In the experiments, the inertial cavitation threshold was determined using a narrow-band PCD with a center frequency of 610 ± 20 kHz; activity detected above the noise floor in this frequency band was assumed to be broadband emissions caused by inertial cavitation. The spatial sensitivity of the transducer was simulated by modeling it as a transmitter. Figures 10(a) and 10(b) show the pressure field radiated by the PCD at 610 kHz. The maximum sensitivity of the detector was in the near-field of the FUS beam for the target sonicated in the experiment (“+” symbols in Fig. 10), and there was substantial spatial variation. We also investigated the sensitivity pattern for subharmonic emission (110 kHz) with this detector. As seen in Figs. 10(c) and 10(d), there was less spatial variation at this frequency, and there was greater sensitivity at the sonicated target.

**FIG. 11.**
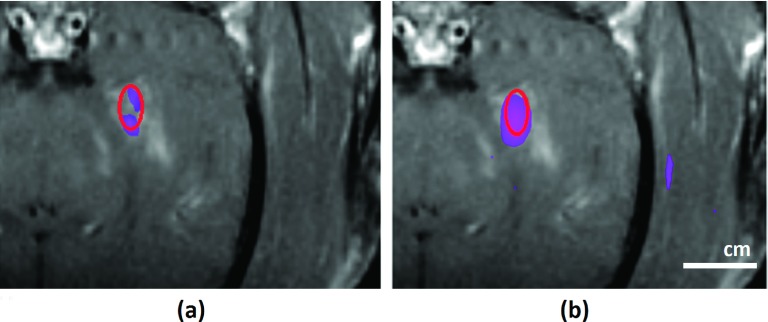
PCD+FUS intensity pattern (normalized with −6 dB lower threshold) plotted on T1 weighted contrast images (a) for 610 kHz, (b) for 110 kHz frequency. The −6-dB intensity for PCD+FUS intensity for a uniform PCD pattern is outlined as an ellipse.

The spatial heterogeneity of the PCD reception field suggested a potential limit on the sensitivity and spatial acuity of the sensor. In order to examine the region of sensitivity inside the cavitation region, the acoustic field of the FUS transducer was multiplied by the acoustic field of the PCD (denoted here as PCD+FUS). In Fig. 11, normalized PCD+FUS maps for 610 and 110 kHz are shown with a −6-dB threshold applied together with the −6-dB FUS beam contour. In the 610 kHz case, the combined simulations suggest that the PCD was not sensitive to the cavitation activity at the center of the beam volume, whereas for the 110 kHz case, the sensitivity profile covered most of the FUS focus. The intersection region of the −6-dB FUS+PCD pattern inside the −6-dB FUS volume is calculated to be 47%, and 78% for 610 and 110 kHz frequencies, respectively.

## 4. DISCUSSION

The previously described experiments^23^ found that it was possible to combine FUS and a microbubble ultrasound contrast agent to ablate deep targets near the skull base while avoiding the skull heating that currently limits thermal ablation to centrally located targets in the brain.^3,11^ In addition to the lesions, we observed BBB disruption, and in some cases, damage in the prefocal region of FUS beam path. The simulations performed in this study aimed to understand these side-effects. We considered several factors, including skull-induced aberration, reflections from the skull base, nonlinear propagation, and distortions induced by beam steering. We also investigated whether improper placement of our PCD’s led to our overestimating the inertial cavitation threshold, leading to our using excessive exposure levels.

The pressure field simulation results showed good agreement with the BBB disruption patterns evident in the T1-weighted contrast-enhanced MRI obtained in the experiment. In particular, the simulations reproduced the pattern of enhancement in the focal area and at the side lobes in the prefocal region. The results from four sonication points simulated for the two animals suggest that the BBB disruption threshold ranged between 74 and 99 kPa. Performing a similar comparison between the simulated field and the hemorrhagic lesion evident in T2*-weighted imaging suggests that the ablation threshold was approximately 277 kPa, about more than three times greater than what was required for BBB disruption. Results from a different study in monkeys with this device^52^ estimated that the probability for MRI-evident BBB disruption and vascular damage was 50% at peak pressures of 149 and 300 kPa, respectively. The ablation threshold calculated in this study is consistent with these results, whereas a lower threshold was found for the BBB disruption. A lower BBB disruption threshold may have been due to using a longer sonication time and a higher microbubble dose, both of which can increase the “magnitude” of the disruption^53,54^ and enabled our detection of smaller amounts of MRI contrast extravasation. In addition, our simulations do not take into account the effect of microbubbles, which might change the pressure field in the side lobes and the calculated thresholds.

Unlike in the prefocal region, BBB disruption was not observed at the side lobes in the postfocal region that were predicted by the simulations. This finding may be a result of shielding caused by microbubbles in the prefocal and focal regions.^55^ We also did not observe enhancement in white matter in the prefocal region. This result was probably due to white matter’s lower vascular density, which results in less extravasated MRI contrast agent after BBB disruption.^52^ For more realistic simulation studies, the distribution of microbubbles in different tissues and their effects on the acoustic propagation should be taken into account. It might also be important to include differences in microbubble behavior in small and large blood vessels that has been shown earlier.^56,57^

For the simulation studies of deep brain targets, the elastic wave model should more accurately model the physical phenomena than an acoustic wave model, since shear mode conversion and absorption is taken into account. Here, 11% difference was observed in the peak pressure for the two models, suggesting that the faster acoustic model may be appropriate to estimate the pressure amplitude, at least with this low-frequency device and for nonhuman primates. Nevertheless, the side lobe pattern was found to be different in acoustic and elastic simulations due to shear mode conversion effects. The simulations also suggest that steering the focal region away from the geometric center may have also increased the size of the side lobes and the extent of the prefocal effects.

The simulations suggest that skull-induced aberration did not have a substantial effect on the dimensions of the main focal zone at 220 kHz, presumably because of the relatively lower phase aberration at this frequency and small thickness of the macaque skull compared to the wavelength of this FUS device. However, it did appear that the presence of the skull increased the amplitude and the volume covered by the first side lobes. Significant effects stemming from signal reflection within the skull (e.g., standing waves) were not observed, despite the sonication targets being only 9 mm away from the skull base. This result is likely due to the high geometric gain of the transducer and is consistent with the previous reported results in which standing waves were not observed for a human skull base target when the full aperture of the hemispherical array was used.^13^

The presence of tissue damage in the prefocal region just in front of focal region could have been due to an overestimation of the inertial cavitation threshold due to suboptimal placement of the PCD’s. The sensitivity map of the PCD’s at the frequency used to detect broadband emissions (610 kHz) had substantial spatial variability. Ideally, a cavitation detector’s sensitivity should be homogeneous throughout the intracranial space. A lower frequency transducer would provide a more spatially homogeneous response with less attenuation, and hence, a reduced sensitivity to tissue inhomogeneity.

Simulation studies can be useful for predicting the pressure distribution inside the skull and possible side effects prior to transcranial MR-guided FUS therapy. These simulations require a CT image of the head with a predicted placement of the skull inside the therapy device. While the simulations require additional time, they can provide a means of assessing the outcome of the procedure and predicting possible safety concerns. In addition, given that the usage of PCD’s is essential to control the procedure for applications involving microbubbles, the placement of the PCD becomes critical since it affects the spatial sensitivity inside the skull. If a moveable PCD were used, it is important to optimize its placement and trajectory using patient-specific simulations. This would require computationally expensive simulations; therefore, fast methods such as ray tracing may be more appropriate. Inaccuracies in the placement and alignment of PCD’s according to simulation results can become a hindrance in the practical application of preprocedural simulations. Alternatively, a fixed receiver array on the same half-spherical surface with the transmit array can be used to monitor the cavitation activity with the cost of increased hardware complexity.^28,29^ Using such an array has advantages in terms of spatial resolution and increased sensitivity.

## 5. CONCLUSIONS

Nonthermal ablation using microbubble-enhanced FUS is a promising noninvasive alternative to surgical resection. Since this approach does not cause significant skull heating, it has the potential to increase the treatment envelope where FUS ablation can be used in the brain. In this study, experimental data obtained during nonthermal ablation in a nonhuman primate model were explored with a comparative analysis of experiments and full-wave simulations. It was shown that the pressure patterns obtained using the simulations were consistent with the prefocal beam effects evident in the experiments as MRI-evident BBB disruption. While the simulated pressure field at the brain targets was similar to that in water at the geometric focus, a number of factors appeared to increase the size of the side lobes, including aberration through the skull, steering the focal point, and perhaps shear mode conversion. Reflections from the skull base and nonlinear propagation did not appear to have a substantial effect on the field. Methods to minimize the side lobes would be beneficial in order to minimize effects in the prefocal region and should be investigated in further studies. The experimental data did not agree in the postfocal region, where the simulations suggest that there should have been tissue effects like in the prefocal zone. Including the effects of bubble activity and vascularization may help to explain this discrepancy. Small areas of tissue damage observed in the experiments a few millimeters in front of the focal region may have been due to incorrect alignment of the PCD’s and overestimation of the inertial cavitation threshold. Use of lower-frequency receivers may improve the detection performance by having a more uniform spatial distribution and increased sensitivity in the focal region. PCD sensitivity can be optimized for a specific target location using simulations.

## Figures and Tables

**TABLE I.** Parameters used in the simulations.

Speed of sound (m/s)		Absorption [dB (MHz cm)^−1^]		Density (kg/m^3^)	
*c*_water_	1480	$\alpha \underset{\mathrm{skull},c}{\min},\;\alpha \underset{\mathrm{skull},c}{\max}$	0.64, 25.46	*ρ*_bone_	2200
*c*_bone,*c*_	3100	*α*_brain_	0.34	*ρ*_brain_	1030
*c*_brain_	1560	*α*_water_	2.5 × 10^−5^	*ρ*_water_	1000

**TABLE II.** Comparison of simulation models.

			Volume above different normalized intensity levels^a^ (mm^3^)			
Configuration	Normalized peak pressure	Relative beam peak position (mm)	−3.5 dB	−7 dB	−12 dB	−14 dB
No NHP (elastic simulation)	1	0	36	103	487	1078
Elastic simulation	0.60	1	37	113	638	1272
Acoustic simulation	0.68	0.8	36	109	641	1536
Elastic simulation (no skull base)	0.57	1	44	127	738	1471

^a^ Normalized to beam peak.

**TABLE III.** Normalized output power and peak pressure for different type of aberration correction schemes.

Configuration	Normalized peak pressure at the intended focus	Volume above −3.5 dB^a^ level (mm^3^)	Volume above −14 dB^a^ level (mm^3^)
Water	1	38	1123
NHP (no correction)	0.60	37	1272
NHP (phase correction)	0.63	37	1071
NHP (phase and amplitude correction)^b^	0.51	38	1320
NHP (phase and inverse amplitude correction)^b^	0.66	37	1106
NHP (no correction-target at the geometric focus)	0.68	37	1115

^a^ Normalized to beam peak.

^b^ Total output power of the array kept constant.
